# Active Thermal Sensing for Bonding Structure Damage Detection of Hidden Frame Glass Curtain Wall

**DOI:** 10.3390/s18113594

**Published:** 2018-10-23

**Authors:** Xiaobin Hong, Jinfan Lin, Yuan Liu, Weiying Xu

**Affiliations:** School of Mechanical & Automotive Engineering, South China University of Technology, Guangzhou 510641, China; 201430113067@mail.scut.edu.cn (J.L.); 15270833143@163.com (Y.L.); xuwy@midea.com (W.X.)

**Keywords:** bonding structure damage, active thermal sensing, heating source, fusion enhancement, thermal wave testing

## Abstract

Adhesive bonded structure damage of hidden frame glass curtain wall will cause falling glass, which threatens the security of people and property. Therefore, the damage detection of the adhesive bonded structure of glass curtain wall has great significance. In this paper, active thermal sensing technology for bonding structure damage detection was introduced. Firstly, the thermal wave propagation of bonded structure was analyzed. Second, the simulated annealing algorithm and TracePro simulation were utilized to design the heat source. Thirdly, the platform of active thermal sensing was built, and experiments were conducted. Finally, image fusion enhancement of Laplacian pyramid was utilized to the enhancement process of thermal images. The simulation results showed that the irradiance of the cross-optimization was more uniform, and the uniformity was 17.50% higher than the original design value. The experiments results showed that defects of different sizes and depths can be distinguished. The gray differences of the damages on the depth of 0 mm and 4 mm were 0.025 and 0.045, respectively. The thermal wave testing can detect damage intuitively and rapidly, which is significant for the future research of unmanned detection of bonding structure damage of hidden frame glass curtain wall.

## 1. Introduction

Hidden framing glass curtain wall is generally used in glass buildings, due to its architecturally exterior and excellent seismic performance. However, safety problems threaten the security of people and property, such as falling glass. Due to the abominable installation environment, the adhesive bonded structure has various types of damage, such as aging and cracking, which will develop into debonding. The mechanical balance of glass curtain wall with debonding defect is broken and the glass is easy to fall off. Therefore, the damage detection of the adhesive bonded structure has great significance for ensuring the safety of hidden frame glass curtain wall.

Researches on adhesive bonded structure damage detection of in-service glass curtain wall mainly used dynamic methods [1,2,3,4,5,6,7]. For example, Miao Y. et al. provided a method based on FFT power spectrum of pulse transient dynamic response to study the relationship between the proportion of power spectral main peak frequency and the damage length of the bonded structure in hidden frame supported glass curtain wall [1]. Gu J. et al. used Hilbert-Huang transform to identify the silicone sealant damage of the glass curtain wall by analyzing the vibration transmissibility of vibration response signal IMF [2]. Liu X. et al. proposed a method by using the dynamic measurement of the natural frequency to evaluate the damage extent of curtain wall glass [3]. Hong X. et al. used nonlinear ultrasonic lamb wave and nonlinear ultrasonic modulation respectively to detect debonding defects in hidden frame supported glass curtain walls [4,5]. Xu B. et al. used vibration displacement measurement in damage detection for a frame structure model [8], and used wavelet packet analysis in debonding detection [9,10]. Sethi V. et al. studied vibration control of model frame structure [11,12]. In addition, adaptive algorithm [13], special sensor [14] and data fusion [15] had been used in health monitoring of high-rise structure. The researches of dynamic measurement provided a practical direction for the detection of in-service glass curtain wall. However, dynamic method requires vibrating glass wall, which may cause the glass with adhesive bonded structure damage to fall off.

As a nondestructive sensing technique, thermal wave sensing has advantages of non-contact, fast and effective. According to whether external thermal excitation sources are needed, thermal wave sensing can be divided into passive thermal wave sensing and active thermal wave sensing [16]. Active thermal wave sensing is mainly used for the detection of debonding of composite materials and surface defects of parts when the thermal wave of tested objects is equivalent to the surrounding environment. There are few studies focused on adhesive bonded structure damage detection of hidden framing glass curtain wall using thermal method. Researches on active thermal wave sensing for damage detection include thermal excitation and thermal image enhancement. In the studies of thermal excitation, different thermal excitation sources and thermal excitation functions are used to detect specific objects. The normal thermal excitation sources used in thermal wave testing include flash lamp [17], light emitting diode (LED) [18], terahertz [19], microwave [20]. For example, Yang Z. et al. improved the uniformity and energy utilization of pulsed flash lamp excitation by fabricating cover and reflector [17]. Pickering S.G. et al. used high-power LED arrays as thermal excitation for long pulsed and lock-in thermal excitation to detect carbon fiber-reinforced plastic with artificial defects [18]. Pulsed thermal excitation [21], pulsed phase thermal excitation [22], lock-in thermal excitation [23] and modulation thermal excitation [24,25] are commonly used as thermal excitation functions. For example, Tao N. et al. detected the glue faults between supporting spars and glass fiber-reinforced plastic shells with different thickness by using pulsed thermography [21]. Brown J. et al. studied and compared the phase images of lock-in thermal excitation and constant step thermal excitation on detection of fiber-reinforced plastic strengthened bridge [23]. Guo X. et al. carried out modulated infrared thermal wave nondestructive testing for cladding debonding detection of solid rocket motors by finite element method [24]. A variety of thermal excitation sources are available in thermal wave testing, but have disadvantages of uniformity of thermal excitation and high-power consumption. By optimizing the thermal excitation source, the uniformity of thermal excitation and power consumption can be improved. For different objects, different thermal excitation functions should be applied. For bonded structures, pulsed thermal excitation is suitable for its simple thermal image sequence processing.

In the researches of thermal image enhancement, conventional image processing technologies have been applied to thermal image enhancement, such as principal component analysis [26], signal reconstruction [27], thermographic signal reconstruction (TSR) [28,29], wavelet transform [30] and adaptive image enhancement [31]. For example, Zheng K. et al. proposed a mathematical morphology for the analysis of geometrical structures to highlight the defects of carbon fiber reinforced plastics by subtracting backgrounds [26]. Shepard S.M. used a logarithmic function to fit the temperature curve and reconstructed temperature curve to reduce the influence of non-uniform emissivity [27]. Chapuis B. quantitatively assessed the improvement of the detectivity of defects in smart composite repair patch using TSR approach [28]. Wang J. et al. put forward to an image denoising method based on wavelet transform to reduce the noise of thermographic data [30]. Wu Y. et al. proposed an adaptive thermal image enhancement method based on contourlet transform and adaptive chaotic variation particle optimization to suppress noise and enhance details [31]. In conclusion, these conventional image processing technologies get great results on the improvement of contrast ratio and highlighting defect of thermal image. But, there is still little research of thermal image enhancement on adhesive bonded structure damage of glass materials.

Thermal wave testing uses a thermal excitation source to heat the object to be inspected. The damaged region forms a significant temperature difference with the normal region. The application of active thermal sensing to damage detection has advantages of intuitive, reliable, fast and effective. Taking electromagnetic radiation as heat source has advantages of non-contact, good uniformity, and a large detection area, which is suitable for adhesive bonded structure of glass curtain wall. In this paper, the active thermal sensing is used to detect adhesive bonded structure damage. A mid and far infrared (MFI) thermal excitation device with pulse function is designed and optimized for the testing of adhesive bonded structure damage. Moreover, image fusion enhancement is used to highlight the defects on thermal images.

This paper is organized as follows. Firstly, it introduces the principle of thermal wave testing on glass curtain wall and image fusion enhancement of Laplacian pyramid. Secondly, it introduces the design of thermal excitation. Thirdly, an experiment platform is set up and the experimental results are presented. Finally, the conclusion is given.

## 2. Mechanism and Methodology

### 2.1. Principle of Active Thermal Sensing on Glass Curtain Wall

Most glasses of curtain wall have excellent absorption capacity in mid-infrared radiation segments (3~6 μm) and far-infrared (6~15 μm) radiation segments. The MFI radiation is mostly absorbed by the surface of the glass, which can be seen as a gray body with constant emissivity. Therefore, MFI radiation is selected as heat source of the thermal wave detection, and glass is simplified as a gray body in this paper. After glass curtain wall absorbs MFI radiation, the temperature of glass surface rises first. The spectral radiance *E_bλ_* of glass curtain wall can be calculated according to the Planck’s law [32]:

(1) $$E_{b\lambda }=\varepsilon_{g}\frac{C_{1}\lambda^{-5}}{e^{C_{2}/(\lambda T)}-1},$$

where *T* represents the absolute temperature; *λ* is the radiation wavelength; *C*_1_ and *C*_2_ represent the Planck’s first and second constant, respectively; *ε_g_* is the curtain wall glass emissivity. The full-wavelength integration of *λ* on both sides of Equation (1) reveals the relationship between the irradiance of glass and absolute temperature as follow:

(2) $$E_{b}=\int_{0}^{\infty }E_{b\lambda }d\lambda =\int_{0}^{\infty }\varepsilon_{g}\frac{C_{1}\lambda^{-5}}{e^{C_{2}/(\lambda T)}-1}d\lambda =\varepsilon_{g}\sigma T^{4},$$

where *σ* is the Stephen Boltzmann constant.

Equation (2) shows that the temperature of curtain wall glass will rise after receiving thermal excitation radiation, and the irradiance of glass is proportional to the fourth power of the absolute temperature. Thus, the temperature of glass can be detected by using infrared camera to capture the irradiance. Compared with contact testing, thermal wave testing has the advantages of no influence on the surface temperature distribution of the glass, short response time and being suitable for large testing area, which greatly improve the efficiency of damage detection.

**Figure 1:**
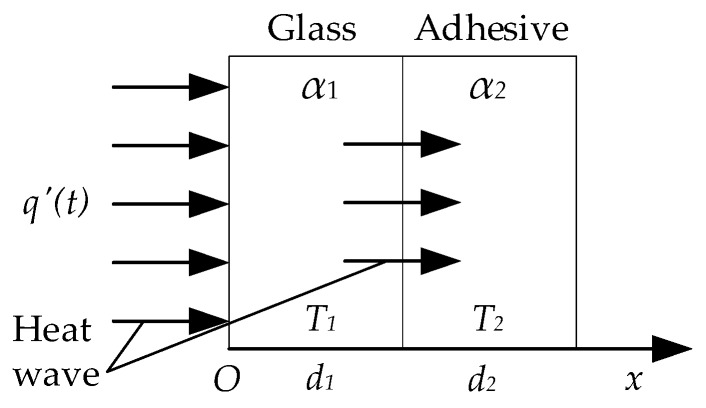
The propagation of heat wave in adhesive bonded structure of glass curtain wall.

MFI radiation is mostly absorbed by the surface of the glass when it irradiates on the glass curtain wall, causing the temperature rising of the glass surface. Then, heat transfers to the interior of the glass in the form of direct thermal wave. The glass curtain wall can be approximated as an infinitely large multi-layer board without an internal heat source. In the thermal wave testing for adhesive bonded structure of glass curtain wall, the propagation of heat wave in adhesive bonded structure of glass curtain wall can be regarded as a one-dimensional unsteady heat conduction without inner heat source (Figure 1). The differential equations of one-dimensional unsteady heat conduction are shown in Equation (3):

(3) $$\{\begin{array}{c} \alpha_{1}\frac{\partial^{2}T_{1}\left(x,t\right)}{\partial x^{2}}=\frac{\partial T_{1}\left(x,t\right)}{\partial t}\\ \alpha_{2}\frac{\partial^{2}T_{2}\left(x,t\right)}{\partial x^{2}}=\frac{\partial T_{2}\left(x,t\right)}{\partial t} \end{array},$$

where *α*_1_ and *α*_2_ are the Stephen Boltzmann constant.

During the thermal wave testing of glass curtain wall, the power of thermal excitation source is far greater than the power loss to the environment. Ignoring the heat conduction of the environment and the glass curtain wall, the glass is only affected by the MFI radiation source. The thermal excitation can be express by the following equation:

(4) $${-\lambda_{1}\frac{\partial T_{1}\left(x,t\right)}{\partial x}|}_{x=0}=q\left(t\right)=\alpha_{0}q^{\prime }\left(t\right),$$

where *λ*_1_ is the thermal conductivity of the curtain wall glass; *q′*(*t*) is the heat flux density of the thermal excitation source, also known as irradiance; *α*_0_ is the absorption rate of glass; *q*(*t*) is the irradiance absorbed by the glass.

The glass is tightly bonded by the structural adhesive. Therefore, the thermal parameters on both sides of the bonding interface are continuous. The continuous conditions are shown as follows:

(5) $$T_{1}\left(d_{1},t\right)=T_{2}\left(d_{1},t\right),$$

(6) $${-\lambda_{1}\frac{\partial T_{1}\left(x,t\right)}{\partial x}|}_{x=d_{1}}{=-\lambda_{2}\frac{\partial T_{2}\left(x,t\right)}{\partial x}|}_{x=d_{1}},$$

where *λ*_2_ is the thermal conductivity of structural adhesive; *d*_1_ and *d*_2_ are thicknesses of glass and adhesive, respectively.

Setting the temperature of glass surface is *T*_0_.

(7) $$T_{1}\left(x,0\right)=T_{2}\left(x,0\right)=T_{2}\left(\infty ,t\right)=T_{0},$$

(8) $${\bar{T}}_{2}\left(\infty ,s\right)=0.$$

To simplify the subsequent analysis, detection temperature scale *T*_0_ = 0 was used in thermal wave testing. The final temperature distribution of the glass needs to add the true temperature of the glass curtain wall.

By using Equation (3) into (7), the one-dimensional unsteady Laplace equations can be obtained as follow:

(9) $$\{\begin{array}{c} \alpha_{1}\frac{\partial^{2}{\bar{T}}_{1}\left(x,s\right)}{\partial x^{2}}=s{\bar{T}}_{1}\left(x,s\right)\\ \alpha_{2}\frac{\partial^{2}{\bar{T}}_{2}\left(x,s\right)}{\partial x^{2}}=s{\bar{T}}_{2}\left(x,s\right) \end{array}.$$

The boundary conditions are shown as follows:

(10) $${-\lambda_{1}\frac{\partial {\bar{T}}_{1}\left(x,s\right)}{\partial x}|}_{x=0}=Q\left(s\right),$$

(11) $${-\lambda_{1}\frac{\partial {\bar{T}}_{1}\left(x,s\right)}{\partial x}|}_{x=d_{1}}{=-\lambda_{2}\frac{\partial {\bar{T}}_{2}\left(x,s\right)}{\partial x}|}_{x=d_{1}},$$

(12) $${\bar{T}}_{1}\left(d_{1},s\right)={\bar{T}}_{2}\left(d_{1},s\right)$$

where $\bar{T_{1}}$(x, s)$,\;\bar{T_{2}}$(*x*, *s*) and *Q*(*s*) are the Laplace transforms of *T*_1_(*x*, *t*), *T*_2_(*x*, *t*) and *q*(*t*), respectively.

**Figure 2:**
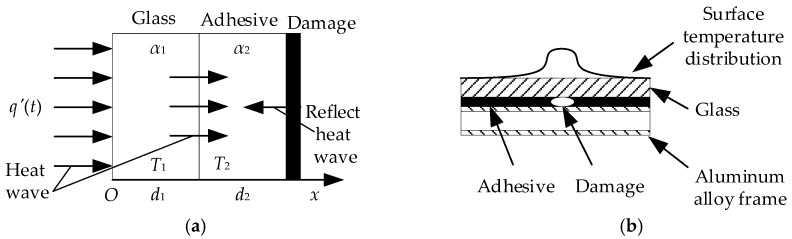
Thermal wave propagation and temperature distribution of damaged bonded structure. (**a**) Heat wave propagation in damaged bonded structure; (**b**) temperature distribution of damaged bonded structure.

When there is a damaged in adhesive bonded structure, the heat wave is blocked to the deep layer of the structural adhesive. Reflected heat wave is formed at the damage interface, which is reflected again when it propagates to the surface of the glass [33]. As shown in Figure 2, the thermal wave is composed of direct thermal wave from the thermal excitation and reflected heat wave, causing the temperature of glass rising. Due to the rapid attenuation of reflection thermal wave, the reflected heat wave on the surface of glass is neglected. A heat conduction differential equation system of reflected heat wave, which is similar to the Equations (3) to (7), can be constructed. The derivation process is omitted. However, it will cause complex formulas and solving processes. The system theory is utilized to simplify the process. Analyzing the differential equations in the *s*-domain, theoretical relationships can be obtained as follow:

(13) $$\{\begin{array}{c} {\bar{T}}_{1}\left(x,s\right)=Q\left(s\right)G_{1}\left[h_{1}\left(\alpha_{1},\alpha_{2},\lambda_{1},\lambda_{2}\right),f_{1}\left(d_{1},\infty \right),x,s\right]\\ {\bar{T}}_{2}\left(x,s\right)=Q\left(s\right)G_{2}\left[h_{2}\left(\alpha_{1},\alpha_{2},\lambda_{1},\lambda_{2}\right),f_{2}\left(d_{1},\infty \right),x,s\right] \end{array},$$

where *G*_1_ and *G*_2_ are functions of heat conduction system of glass and structural adhesive, respectively. *h*_1_ and *h*_2_ are materials modulation functions of glass and structural adhesive, respectively. *f*_1_ and *f*_2_ are structure modulation functions of glass and structural adhesive, respectively.

The infrared (IR) camera can only capture the surface temperature of the glass. The transfer function of the glass surface temperature $\bar{T_{1}}$(*0*, *s*) is called the thermal response of glass, showed as follow:

(14) $${\bar{T}}_{1}\left(s\right)=Q\left(s\right)G_{1}\left[f_{1}\left(d_{1},\infty \right),s\right].$$

When there is a damaged in adhesive bonded structure, the thermal wave is composed of direct thermal wave from the thermal excitation and reflected heat wave. Therefore, the transfer function of the glass surface temperature with damaged bonded structure can be expressed as follow:

(15) $${\bar{T}}_{d1}\left(s\right)=Q\left(s\right)G_{d1}\left[f_{d1}\left(d_{1},d_{2}\right),s\right],$$

where *G*_d1_ is the function of heat conduction system of damaged adhesive bonded structure. *f_d_*_1_ is the structure modulation function of damaged adhesive bonded structure.

For composite thermal excitation formed by linear superposition of *N*_h_ thermal excitation sources, theoretical relationships can be obtained as follow:

(16) $$\{\begin{array}{c} {\bar{T}}_{1}\left(s\right)=G_{1}\left[f_{1}\left(d_{1},d_{2}\right),s\right]\sum_{i=0}^{N_{h}-1}Q_{i}\left(s\right)=\sum_{i=0}^{N_{h}-1}Q_{i}\left(s\right)G_{1}\left[f_{1}\left(d_{1},d_{2}\right),s\right]\\ {\bar{T}}_{d1}\left(s\right)=G_{d1}\left[f_{d1}\left(d_{1},d_{2}\right),s\right]\sum_{i=0}^{N_{h}-1}Q_{i}\left(s\right)=\sum_{i=0}^{N_{h}-1}Q_{i}\left(s\right)G_{d1}\left[f_{d1}\left(d_{1},d_{2}\right),s\right] \end{array}.$$

The thermal response of composite thermal excitation is a linear superposition of the multiple thermal excitation responses. The Laplace inverse transformation is performed on both sides of the Equation (16). Considering the initial temperature, the actual temperature of glass surface is as follows:

(17) $$\{\begin{array}{c} T_{a1}\left(t\right)=T_{1}\left(t\right)+T_{0}=\sum_{i=0}^{N_{h}-1}q_{i}\left(t\right)\cdot g_{1}\left[f_{1}\left(d_{1},d_{2}\right),t\right]+T_{0}\\ T_{\mathrm{ad}1}\left(t\right)=T_{d1}\left(t\right)+T_{0}=\sum_{i=0}^{N_{h}-1}q_{i}\left(t\right)\cdot g_{d1}\left[f_{d1}\left(d_{1},d_{2}\right),t\right]+T_{0} \end{array},$$

where “*” represents the convolution operation; *q_i_*(*t*) is the impulse response function of the *i*-th thermal excitation; *g*_1_ and *g*_d1_ are the impulse response functions of heat conduction systems of adhesive bonded structure with no-damage and damage, respectively.

For a specific glass curtain wall, the surface temperature of the glass is only related to the thermal excitation, time and structural modulation function. The structural modulation function can be identified from the temperature change of the surface of the glass, thereby making it possible to detect the damage of adhesive bonded structure.

Taking the temperature change of the surface of the glass as a self-reference, the differential operation of the Equation (17) is performed to obtain the surface temperature difference, that is the temperature difference caused by the reflected heat wave.

(18) $$\begin{array}{ll} \Delta T_{1}\left(t\right) & =T_{a1}\left(t\right)-T_{ad1}\left(t\right)\\ & =\left[T_{1}\left(t\right)+T_{0}\right]-\left[T_{d1}\left(t\right)+T_{0}\right]\\ & =\sum_{i=0}^{N_{h}-1}q_{i}\left(t\right)\cdot \left\{g_{1}\left[f_{1}\left(d_{1},d_{2}\right),t\right]-g_{d1}\left[f_{d1}\left(d_{1},d_{2}\right),t\right]\right\} \end{array}$$

The influences of the initial temperature on the detection result can be eliminated while the interference of common mode and noise are reduced after differential operation. Moreover, it is not necessary to use the standard test block for calibration. As for thermal images, the shape of the bonded structure damage can be measured by the different result of each pixel.

### 2.2. Active Thermal Sensing Framework of Damage Detection

The accuracy of identification results of the structure modulation function is closely related to thermal excitation. The bandwidth of the thermal excitation function must be larger than the frequency bandwidth of heat conduction system. Moreover, the optimal excitation function of the thermal excitation is required to be a time domain autocorrelation function with a correlation peak and a salient pulse. To ensure the thermal wave can propagate inside the glass curtain wall sufficiently, continuous excitation or high-power density excitation is adopted. However, excessive time or excessive power density of excitation may cause adhesive bonded degenerative aging, which increases the risk of the curtain wall glass falling. Pulse and white noise are the optimal excitation functions of which the autocorrelation functions have ideal bandwidth and only one prominent main pulse. However, white noise is difficult to generate and its time domain length is less than pulse. Therefore, pulse is selected as the excitation function of the thermal wave testing for adhesive bonded structure of glass curtain wall.

In this paper, carbon fiber radiator is selected as the key component of the thermal excitation device for its high energy density. The radiation segment of carbon fiber radiator covers visible light to far infrared, but the radiation segment of which the wavelength is less than 3 μm can be ignored. Short thermal response time is beneficial to the design of the control system and generation of the pulse thermal excitation function.

**Figure 3:**
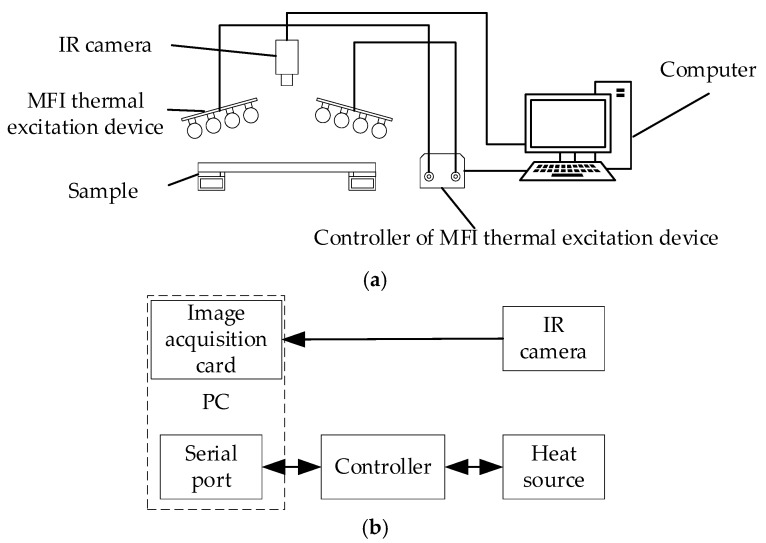
Schematic diagram of active thermal sensing platform for bonded structure damage detection. (**a**) Active thermal sensing platform for bonded structure damage detection; (**b**) framework of active thermal sensing platform for bonded structure damage detection. MFI, mid and far infrared. IR, infrared.

According to the selection of the thermal excitation function and the radiation device, the active thermal sensing platform for adhesive bonded structure damage detection is set up, as shown in Figure 3a, and framework of the platform is shown in Figure 3b.

The pulse thermal excitation function from the MFI thermal excitation device excites the glass curtain wall, causing the surface temperature of damage area higher than the non-damage area. IR camera is used to capture the surface temperature of the curtain wall glass. The information of damage such, as area and depth, can be determined by using image fusion enhancement to analyze the thermal images.

### 2.3. Principle of Laplacian Pyramid Image Fusion Enhancement

The thermal image sequence has a large amount of redundant information and is low contrast. Therefore, a fusion enhancement algorithm of thermal image sequence is put forward to in this paper. First, a Gaussian pyramid is built. Then a Laplacian pyramid is built on the basis of the Gaussian pyramid to separate the shape and detail of the thermal images. Finally, fusion enhancement rules on pixel layer are used to analyze the thermal image sequence.

In Gaussian pyramid decomposition, fuzzy processing and down sampling are used to obtain a series of thermal image sequences with different sizes and clarity, which simulate the detection results of thermal images at different scales and resolutions. The thermal image after pretreatment, named *I*_0_, is placed at the bottom of the Gaussian pyramid. The image at the *l*-th layer is named *I_l_*. A 5 × 5 Gaussian kernel function *W*(*m*, *n*) is used to analyze the image convolution algorithm of the *l*-1-th layer. The results of convolution operation are subjected to down sampling to obtain the thermal images of the *l*-th layer. The number of Gaussian pyramid layers is named *N*. The column number of the *l*-th layer thermal images is named *C_l_*, and the row number of the *l*-th layer thermal images is named *R*_1_.

(19) $$I_{l}=\sum_{m=-2}^{2}\sum_{n=-2}^{2}W\left(m,n\right)I_{l-1}\left(2i+m,2j+m\right),0<l\leq N,0<i\leq C_{l},0\leq j<R_{l}$$

Magnification by interpolation is used on the *l*-th layer images in the Gaussian pyramid to obtain magnified thermal images, which have the same size with the *l*-th layer images. The equations of magnification are shown as follows:

(20) $$I_{l}^{*}=4\sum_{m=-2}^{2}\sum_{n=-2}^{2}W\left(m,n\right)I^{\prime }{}_{l-1}\left(\frac{i+m}{2},\frac{j+n}{2}\right),0<l\leq N,0<i\leq C_{l},0\leq j<R_{l},$$

(21) $$I^{\prime }{}_{l-1}\left(\frac{i+m}{2},\frac{j+n}{2}\right)=\{\begin{array}{lll} I_{l-1}\left(\frac{i+m}{2},\frac{j+n}{2}\right) & , & \frac{i+m}{2}\mathrm{and}\frac{j+n}{2}\mathrm{are}\;\mathrm{integer}\\ 0 & , & \mathrm{other} \end{array}.$$

I_l_^*^ is called the scale operator and is noted as follow:

(22) $$I_{l}^{*}=Expand\left(I_{l}\right).$$

The differences between *I_l_* and I_l_^*^ are calculated to obtain the difference images. A Laplacian pyramid with the same number of layers as the Gaussian pyramid is built by stacking the difference images. The *l*-th layer of the Laplacian pyramid is defined as follow:

(23) $$\{\begin{array}{lll} LP_{l}=I_{l}-Expand\left(I_{l+1}\right) & , & 0\leq l<N\\ LP_{l}=I_{l} & , & l=N \end{array}.$$

Compared with the Gaussian pyramid, the images of the Laplacian pyramid include more details in different dimensions. The process of Laplacian pyramid thermal images separates the shape and details of the thermal images. Fusion enhancement rules include rules based on pixel layer, regional layer and feature layer. The rules of pixel layer are suitable for the images with similar property obtained by the same type of detectors. The pixel layer rules are adopted as the fusion enhancement rules of the thermal image in this paper.

Construct a Laplacian pyramid with *N* layers from the *N_f_* thermal images. The strategy of minimum fusion on pixel layer is used on the *N*-th layer images of the Laplacian pyramid, which reflect the shapes of the thermal images to increase the images contrast. The strategy of maximum fusion on pixel layer is used on the other layer, which reflect the details of the thermal images to highlight the details. *FI_l_* is the *l*-th layer of the fusion enhancement of the Laplacian pyramid. *LP_kl_* is the *l*-th layer of the *k*-th image of the Laplacian pyramid.

(24) $$FI_{l}=\{\begin{array}{lll} \sum_{k=0}^{N_{f}-1}\min\left[LP_{kl}\left(i,j\right)\right] & , & l=N\\ \sum_{k=0}^{N_{f}-1}\max\left[LP_{kl}\left(i,j\right)\right] & , & 0\leq l<N,0<i\leq C_{l},0<j\leq R_{l} \end{array},$$

where min[ ] represents taking the minimum value and max[ ] represents taking the maximum value.

**Figure 4:**
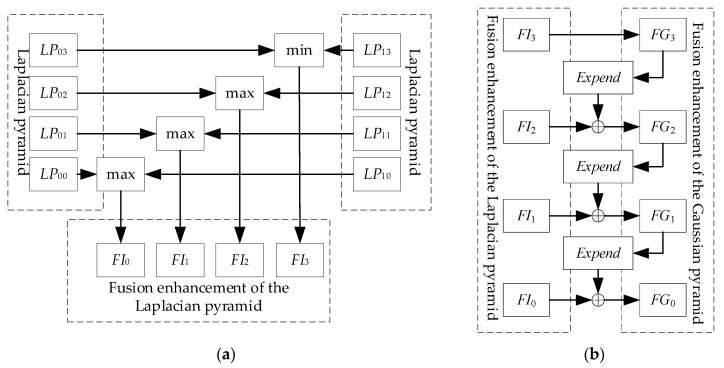
The process of the fusion enhancement. (**a**) The process of Laplacian pyramid; (**b**) the process of Gaussian pyramid.

Figure 4a shows the process of the fusion on pixel layer [34]. The images of each layer of the Laplacian pyramid are subjected to the magnification operation, and then being summed to get a fusion-enhanced Laplacian pyramid, showed as Figure 4b. *FG*_0_ is the result of the fusion enhancement.

## 3. Design of MFI Thermal Excitation

### 3.1. Irradiance Distribution of MFI Thermal Excitation Device

Carbon fiber radiator is selected as the thermal excitation source for its high energy density. In order to get a uniform irradiance of the MFI thermal excitation, multiple carbon fiber radiators are required and their arrangement should be optimized.

**Figure 5:**
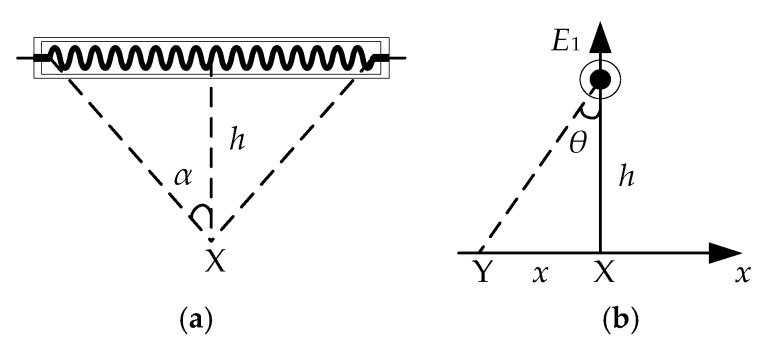
X-point and Y-point of carbon fiber radiator. (**a**) X-point of carbon fiber radiator; (**b**) Y-point of carbon fiber radiator.

As shown in Figure 5a, the irradiance of the X-point can be calculated as follow [35]:

(25) $$E_{0}=\frac{\eta \varepsilon_{g}P}{2\pi^{2}l_{0}h}\left(2\alpha +2\sin2\alpha \right),$$

where η is the conversion rate of electro-thermal radiation; P is the power of carbon fiber radiator; $l_{0}$ is the length of carbon fiber radiator; h is the distance between the carbon fiber radiation source and the glass. As shown in Figure 5b, the Y-point irradiance can be obtained by Equation (26):

(26) $$E_{1}=E_{0}\cos\theta =\frac{\eta \varepsilon_{g}P}{2\pi^{2}l_{0}}\left(2\alpha +2\sin2\alpha \right)\frac{1}{\sqrt{x^{2}+h^{2}}}=K\frac{1}{\sqrt{x^{2}+h^{2}}}.$$

**Figure 6:**
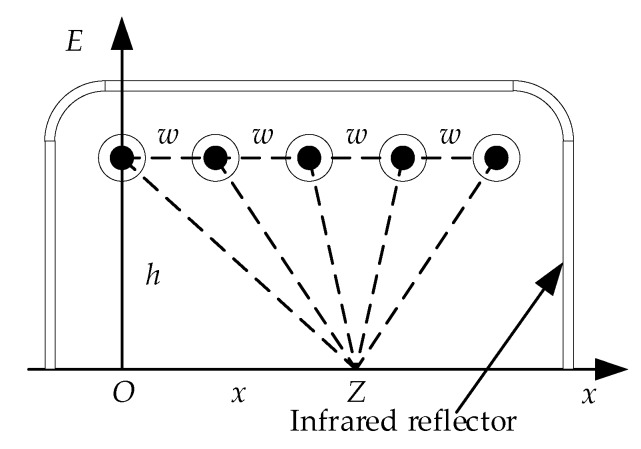
Irradiance of infrared thermal excitation device.

An MFI thermal excitation device formed by $N_{c}$ carbon fiber radiators distributed in parallel is shown in Figure 6. The distance of two adjacent carbon fiber radiators is *w*. Rough infrared reflector is produced by sand-blasting process of quartz sand on the inner surface of the case to form uniform diffuse reflection of the thermal excitation. The irradiance of the Z point is composed of the reflector reflection and the direct radiation, which can be calculated as follow:

(27) $$E\left(w,h,x\right)=E_{2}+K\sum_{i=1}^{N_{c}}\frac{1}{\sqrt{{\left(x-iw\right)}^{2}+h^{2}}}.$$

In the thermal wave testing for adhesive bonded structure damage of glass curtain wall, the more uniform the irradiance is, the less interference is. The uniform of the irradiance can be improved by optimizing the *w* and the *h.*

To measure the range of thermal excitation, the effective area (EA) of radiation for the infrared thermal excitation device is defined as the maximum distance of the irradiance inflection points. Uniformity (UN) is the standard deviation of the irradiance within the effective range of radiation, which is taken as the index to measure the uniformity.

Let the *h* be a fixed value and the uniformity can be optimized by using simulated annealing algorithm. After optimization, the relationship between w and h can be obtained by a suitable fitting function to get an optimal *w*.

**Figure 7:**
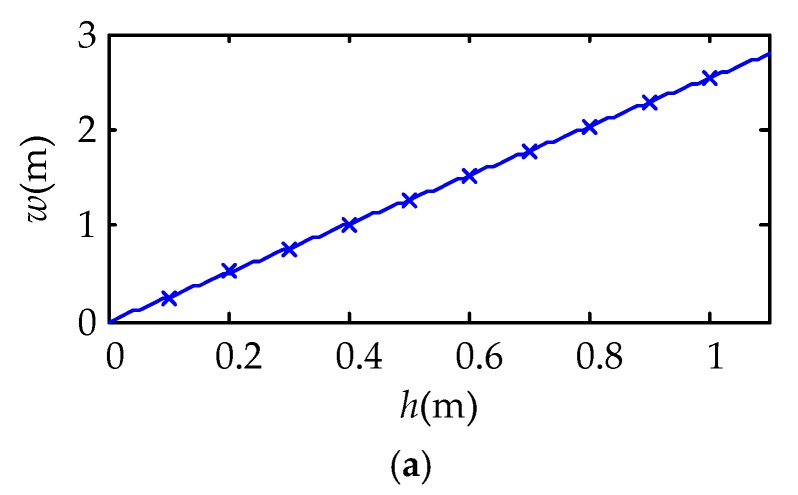

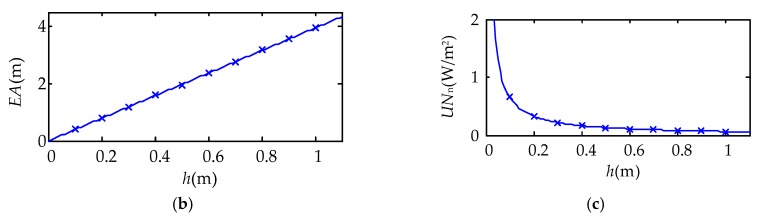
Optimization results of simulated annealing algorithm. (**a**) Optimization results of tube spacing w; (**b**) optimization results of effective radiation range effective area (EA); (**c**) optimization results of normalized uniformity $UN_{n}$.

The number of carbon fiber radiator is 2, and K is normalized to 1W/m. *h* = 0.1, 0.2, …, 1.0. The optimization results are shown in Figure 7.

According to the simulated annealing algorithm result, the relationships in the SI system by curve fitting is obtained as follow:

(28) $$\{\begin{array}{c} w=2.535h\\ EA=3.950h\\ UN_{n}=0.066h^{-1} \end{array}.$$

The optimization results show that the *w* and *h* have obvious linear relationship. From Figure 7, under a rise of *h*, the effective area increases, but the uniformity decreases. In a specific testing, it is necessary to consider the *h* according to the detected object.

### 3.2. Simulation Experiment of Thermal Excitation Source

To verify the effectiveness of the design for thermal excitation source, the design of irradiance distribution is simulated by TracePro 7.0. The parameters of carbon fiber radiators and curtain wall glass are shown in Table 1.

According to the optimization result Equation (28), the optimized parameters of thermal excitation source can be obtained, as shown in Table 2. The simulation parameter settings are shown in Table 3.

**Figure 8:**
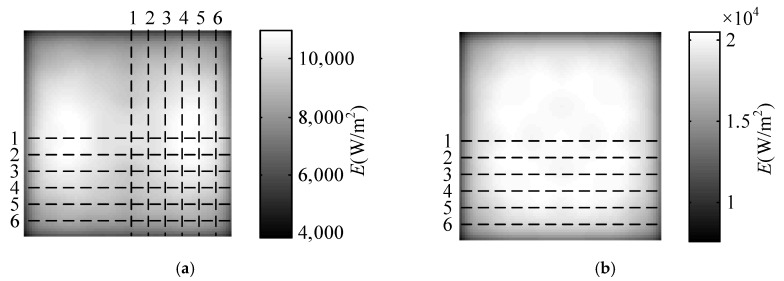
TracePro simulation irradiance distribution map. (**a**) Uncrossed; (**b**) cross-optimization.

**Figure 9:**
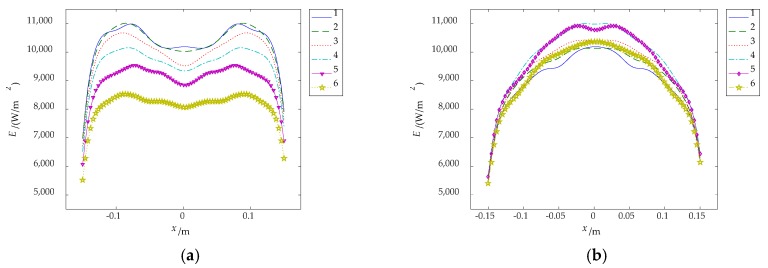
Irradiance distribution of sample line. (**a**) Transverse sampling line; (**b**) longitudinal sampling line.

The irradiance distribution obtained by the simulation is shown in Figure 8a. The irradiance distribution of the sampling lines of Figure 8a are shown in Figure 9. In the irradiance distribution of the transverse sample lines, with the distance between symmetry axis and sample lines increasing, the irradiance decreases. In the irradiance distribution of the longitudinal sample lines, with the distance between symmetry axis and sample lines increasing, the irradiance increases first and then decreases.

**Figure 10:**
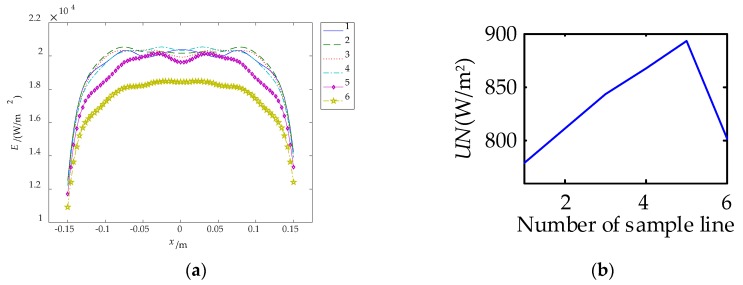
Irradiance distribution and uniformity of sample line. (**a**) Irradiance distribution; (**b**) uniformity.

The irradiance distributions in transverse and longitudinal direction forms an approximate complementary relationship. Therefore, four carbon fiber radiators are arranged in a square shape, called cross-optimization arrangement, and the optimization results are shown in Figure 8b. Six sampling lines of Figure 8b are taken to get the irradiance distribution of the sample lines, as shown in Figure 10.

The uniformity after cross optimization arrangement is 2310 W/m^2^. Considering four carbon fiber radiators, the uniformity of two carbon fiber radiators is 1155 W/m^2^, which is 17.50% higher than the design value. As shown in Figure 10a, the irradiance of the sample line after cross-optimization become more uniform. The irradiance distribution of sample line 1 to line 4 are approximately uniform, which therefore can be taken as detection area.

## 4. Experiments and Results

### 4.1. Experiment Platform

**Figure 11:**
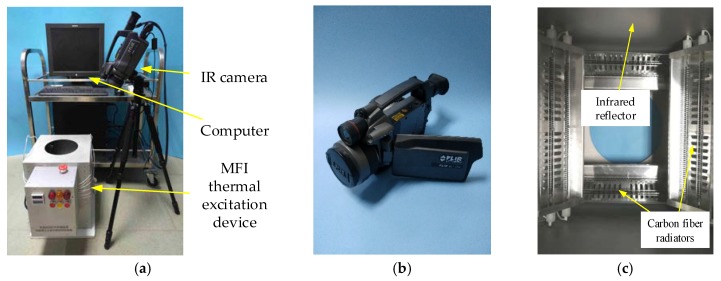
Active thermal sensing platform of glass curtain wall bonding structure damage detection. (**a**) Complete platform; (**b**) thermal imager; (**c**) crossed distribution of carbon fiber radiators.

**Figure 12:**
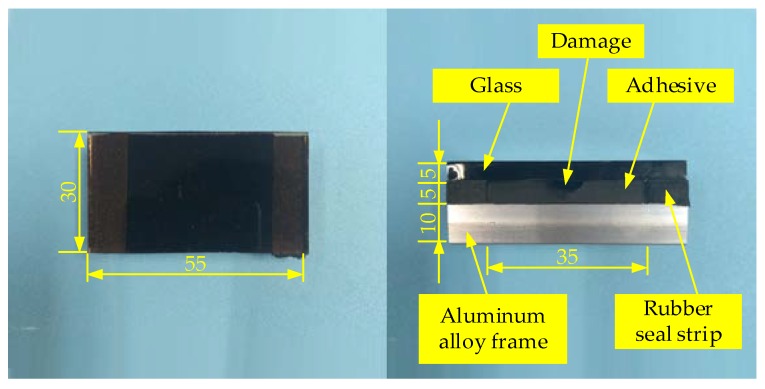
Sample for thermal wave detection of glass-curtain wall bonded structure damage.

An active thermal sensing platform of adhesive bonded structure damage detection of glass curtain wall was built, as shown in Figure 11. The platform is composed of the MFI thermal excitation device, FLIR SC660 IR camera and PC. The MFI thermal excitation device heats the sample and raises the surface temperature of the glass. Thermal IR camera collects the irradiance of the glass surface periodically, and reconstructs the surface temperature in the form of thermal image sequence. Finally, Fusion enhancement algorithm is used to highlight the damage. Figure 11c shows the distributions of carbon fiber radiators. The crossed arrangement can offer a uniform irradiance in central region. The infrared reflector is produced by sand-blasting process of quartz sand on the inner surface to form uniform diffuse reflection of the thermal excitation. The actual size of the curtain wall glass is great. To meet the requirements of laboratory research, glass curtain wall samples in a size 30 × 55 mm are produced, as shown in Figure 12.

### 4.2. Experiment of Damage Detection

The high-power MFI thermal excitation source in this paper can increase the temperature difference between the damage and non-damage area of the glass surface. To Verify the effect of active thermal sensing platform, the experiments of the damage detection for adhesive bonded structure of the glass curtain wall were carried out.

**Figure 13:**
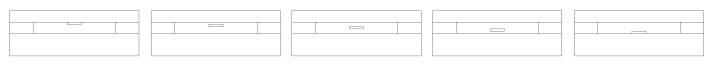
Depth of damages location.

Five samples with same damages were taken in the experiments. The width of damages is 6 mm, and length is 30 mm, and thickness is 1 mm. The depths of damages locations were 0 mm, 1 mm, 2 mm, 3 mm and 4 mm, respectively (Figure 13). The 0 mm damage is surface damage of adhesive and the others is internal damages. In the experiments, sunshine was taken as a contrast to the MFI thermal excitation source. Environmental data is shown in Table 4.

**Figure 14:**
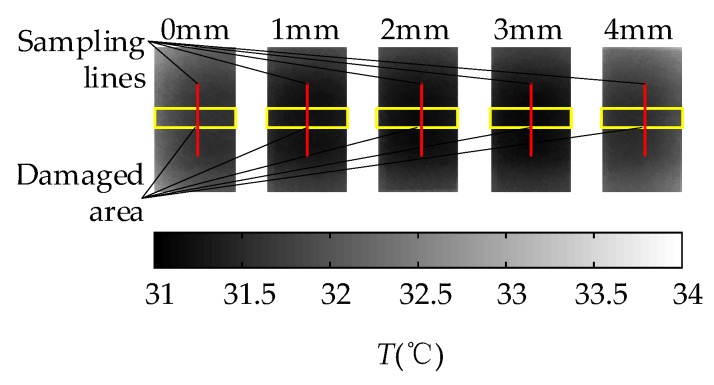
The thermal image of 30 min of sunshine excitation.

**Figure 15:**
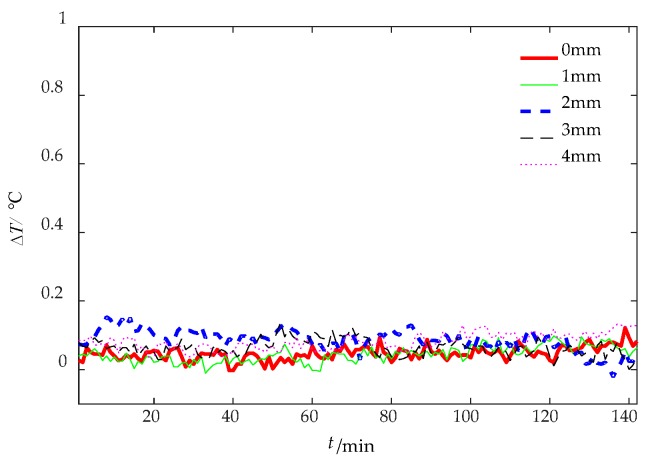
The surface temperature difference curves of sunshine excitation.

In the comparative experiment, the sampling rate of the IR camera was one frame per minute and the sampling time was 142 min. The thermal image of 30 min is shown in Figure 14. As Figure 12, both sides of damage area are the non-damaged area. The central axis was taken as sampling line and the temperature difference is obtained by subtracting the maximum and minimum of the sampling line. The curves of surface temperature differences between damaged area and non-damaged area are shown in Figure 15. The difference of grayscale between damaged area and non-damaged area is extremely insignificant. Therefore, sunshine is not suitable as heat source in the damage detection.

**Figure 16:**
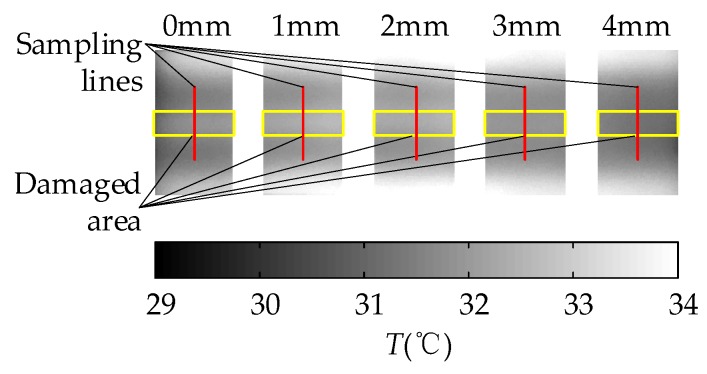
The thermal image of 30 s of the MFI thermal excitation.

**Figure 17:**
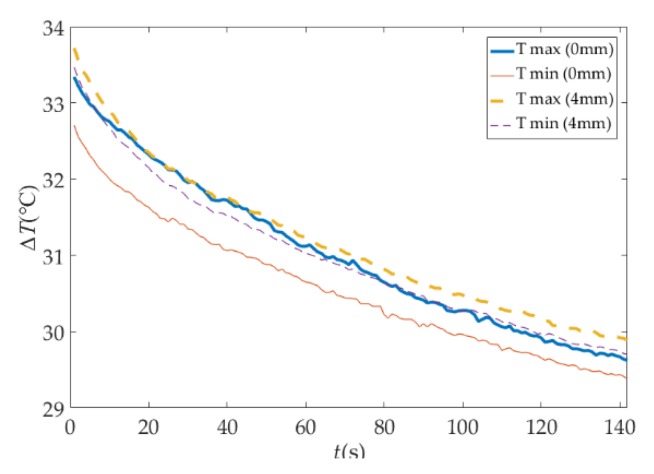
The maximum and minimum temperature of the 0 mm and 4 mm depth damaged.

**Figure 18:**
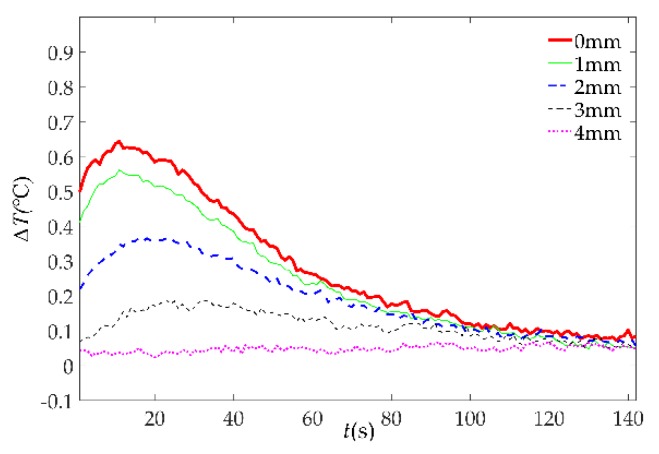
The surface temperature difference curves of the MFI thermal excitation.

**Figure 19:**
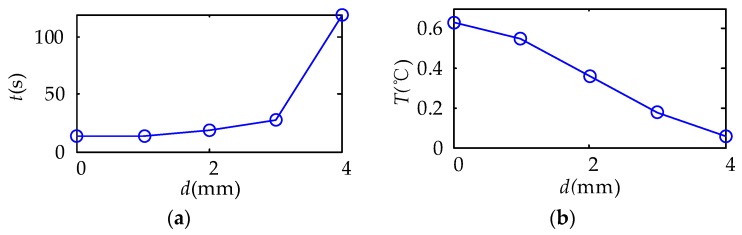
The relationships between the surface temperature difference curves and the depth of damages location. (**a**) The relationships between time of maximum surface temperature difference and the depth of damages location; (**b**) the relationships between maximum surface temperature difference and the depth of damages location.

In the experiment of the MFI thermal excitation, the thermal excitation time was 20 s and then the MFI thermal excitation device was removed. The sampling rate of the IR camera was 1 frame per second and the sampling time was 142 s. The thermal image of 30 s is shown in Figure 16. The defects can be distinguished on the thermal image, for the differences in gray scale at defects are apparent. With the depth of damages increases, the color of the damaged area becomes darker and the contrast between damaged area and non-damaged area becomes smaller. But the effect of deep defect (more than 4 mm) is not obvious. The maximum and minimum temperature of the 0 mm and 4 mm depth damaged are showed in Figure 17. The curves of surface temperature difference between damaged area and non-damaged area are shown in Figure 18. The surface temperature differences are in the range of 0.02 °C to 0.64 °C. The curve of 0 mm increases to the apex and decreases in higher rates compared with the deeper damages. The maximum temperature difference appears near 15 s. Therefore, the temperature difference of 15 s can be used as an index to measure damage. The relationships between the surface temperature difference curves and the depth of damages location are shown in Figure 19. The maximum value of the temperature difference has an approximate linear relationship with the depth of the damage location. The maximum temperature difference of 0 mm is 0.64 °C while the 4 mm is smaller than 0.1 °C. With the depth of damages location increasing, the time of the maximum value of the temperature difference is delayed, and the maximum value is lower.

When there is a damage in adhesive bonded structure, the heat wave is blocked to the deep layer of the structural adhesive. Reflected heat wave is formed at the damage interface and propagates to the glass surface leading to the temperature difference with non-damaged area. With the depth of damage increasing, there is less reflected heat wave propagating to the glass surface and the temperature difference becomes smaller. In conclusion, under the MFI thermal excitation, with the depth of damage increasing, the surface temperature difference between damaged area and non-damaged area decreases.

### 4.3. Experiment of Image Fusion Enhancement

**Figure 20:**
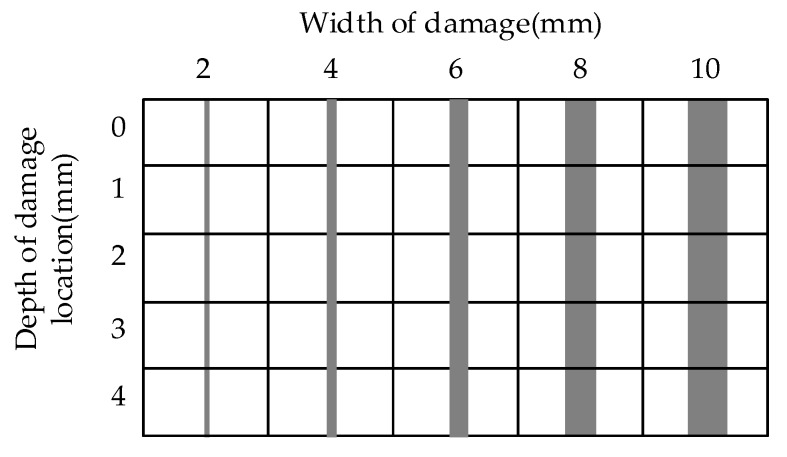
The distribution of samples.

In the fusion enhancement, the more the Laplacian pyramid decomposition layers of the thermal image are, the more thorough separation of detail and shape will be got, and the finer the detail distinction is. Taking the speed of fusion enhancement processing into account, the thermal image is decomposed into four layers in this paper. To speed up the progress of the experiment, the distribution of samples is shown in Figure 20. Each small rectangle represents a sample and the gray area in the rectangle indicates the damage position. The depths of damages are 0 mm, 1 mm, 2 mm, 3 mm and 4 mm, and the widths of damages are 2 mm, 4 mm, 6 mm, 8 mm and 10 mm.

Gray scale of thermal image has a one-to-one correspondence relationship with temperature. The reference of temperature is floating during the enhancement process. To assess the enhancement effect of fusion enhancement, the surface temperature difference is replaced by the gray scale difference.

**Figure 21:**
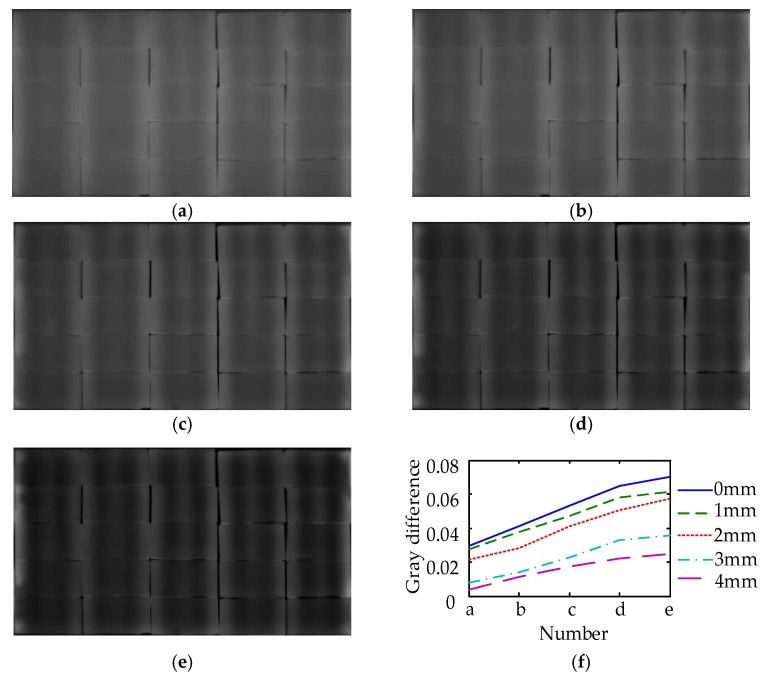
Results of fusion enhancement. (**a**) Results of fusion enhancement (thermal images of 15 s and 15 s); (**b**) results of fusion enhancement (thermal images of 12 s and 18 s); (**c**) results of fusion enhancement (thermal images of 9 s and 21 s); (**d**) results of fusion enhancement (thermal images of 6 s and 24 s); (**e**) results of fusion enhancement (thermal images of 3 s and 27 s); (**f**) relationship between the number of fusion enhancement and the gray scale difference.

Two thermal images are taken as a thermal images-set for image fusion enhancement of the Laplacian pyramid. The thermal images of 15 s and 15 s, 12 s and 18 s, 9 s and 21 s, 6 s and 24 s, 3 s and 27 s are taken as samples in this experiment respectively. The results of the image fusion enhancement of the Laplacian pyramid are shown in Figure 21. With the depth of damages increasing, the contrast of damage gradually decreases. The contrast of damages of 4 mm is small, but can be distinguished by the naked eye. From the results of fusion enhancement, with the time interval of the two thermal images increases, the damages become more obvious. For example, in the fusion enhancement results of the 15 s thermal images, the color of non-damaged area is French grey and the contrast between damaged area and non-damaged area is inconspicuous. In the fusion enhancement results of the 3 s and 27 s thermal images, the color of damaged area is brighter and the color of non-damaged area is darker than the 15 s. Therefore, the contrast is higher and damage is more obvious.

In quantitative analysis of the enhancement effect of the fusion enhancement, the gray differences of damages which is 6 mm in width were taken as examples to obtain the relationship between the number of gray linear transformation and the gray difference. Five relationship curves of different damage depths are shown in Figure 21f. With the time interval of the two thermal images increasing, the gray difference also increases. Comparing the curves of different depths, the increased value of the gray scale differences is similar. For example, the gray differences of the damages at 0 mm depth increases from 0.029 to 0.070 and the growth value is 0.040, while the gray differences of the damages at 4 mm depth increases from 0.004 to 0.025 and the growth value is 0.021.

From the above analysis, the image fusion enhancement increases the damage contrast obviously which is effective for shallow damages and deep damages. Moreover, as the time interval of the two thermal images increasing, the increased values of gray differences are at the same order of magnitude.

## 5. Conclusions

As a nondestructive technology, the active thermal sensing technology is proposed for the damage detection of adhesive bonded structure of glass curtain wall. The effectiveness of the method is verified by studying the principles and experimental verification. Moreover, a special thermal excitation device and the process of image fusion enhancement based on Laplacian pyramid decomposition are carried out to improve the effect of detection.

In the design of MFI thermal excitation device, the optimization results of simulated annealing algorithm show that carbon fiber radiators distance *w* and excitation distance *h* have obvious linear relationship, and under a rise of *h*, the effective area increases, but the uniformity decreases. The uniformity after cross optimization arrangement improves 17.50% comparing with the design value of simulated annealing algorithm. The irradiance of middle area is approximately uniform, which can be taken as detection area. Using the MFI thermal excitation device on experiment, the results showed that the defects can be distinguished on the thermal image, but the effect of deep defect (more than 4 mm) is not obvious. With the depth of damage increasing, there is less reflected heat wave propagating to the glass surface and the temperature difference becomes smaller. The maximum value of the temperature difference has an approximate linear relationship with the depth of the damage location. The maximum temperature difference of 0 mm is 0.64 °C while the 4 mm is lower than 0.1 °C. In the process of image fusion enhancement, with the time interval of the two thermal images increasing, the contrast is higher and damage is more obvious. The fusion enhancement results of 3 s and 27 s thermal images are the optimal in the experiment. The damages can be distinguished by naked-eye, which is effective for shallow damages and deep damages. The gray differences of 6 mm-wide damages are 0.070 at 0 mm and 0.025 at 4 mm. 

The thermal wave testing can detect the damage of the adhesive bonded structure of glass curtain wall directly and visually, which provides a new vision and a new path for further research on damage assessment and online monitoring of glass curtain wall. With the development of the technology, the damage detection system of hidden frame glass curtain wall bonding structure can be mounted on unmanned aerial vehicle or wall climbing robot and used in actual damaged detection in the future.

## Figures and Tables

**Table 1:** Parameters of carbon fiber radiator and curtain wall glass.

Length *l*_0_ (mm)	Diameter *R* (mm)	Power *P* (W)	Electro-Thermal Radiation *η* (%)	Radiant Rate of Glass *ε_g_*
240	10	600	98	0.94

**Table 2:** Optimization parameters of thermal excitation source.

Radiation Distance *h* (mm)	Distance of Adjacent Carbon Fiber Radiators *w* (mm)	Normalized Uniformity *UN*_n_ (W/m^2^)	Uniformity *UN* (W/m^2^)
76	192	0.87	983

**Table 3:** TracePro simulation parameter settings.

Luminous Flux *ψ*(W)	Radiation Type	The Optical Properties of the Inner Surface of the Reflector	The Number of Lights
588	Lambertian	Diffuse reflection	200,000

**Table 4:** Environmental data of sunshine experiment.

Temperature (°C)	Humidity (%)	Weather	Solar Incident Angle (°)
30 ± 0.2	84 ± 5	Sunny day	90
